# Evaluating the Diagnostic Role of the Testosterone-to-Prostate-Specific Antigen Ratio in Pre-Biopsy Risk Stratification of Prostate Cancer

**DOI:** 10.3390/jcm14093022

**Published:** 2025-04-27

**Authors:** Georgios Tsakaldimis, Dimitrios Diamantidis, Stavros Lailisidis, Charalampos Kafalis, Nikolaos Panagiotopoulos, Chrysostomos Georgellis, Stavros Giannopoulos, Chousein Chousein, Marios Spounos, Evangelia Deligeorgiou, Stilianos Giannakopoulos, Christos Kalaitzis

**Affiliations:** 1 Department of Urology, Democritus University of Thrace, 68100 Alexandroupolis, Greece; 2 Department of Pathology, Democritus University of Thrace, 68100 Alexandroupolis, Greece

**Keywords:** prostate cancer, testosterone, prostate-specific antigen (PSA), biomarker, testosterone-to-PSA ratio

## Abstract

**Background:** The testosterone-to-PSA (T/PSA) ratio has been proposed as a novel biomarker to enhance the diagnostic specificity of prostate-specific antigen (PSA) in prostate cancer (PCa) detection. The objective of this study was to evaluate the diagnostic performance of the T/PSA ratio in distinguishing PCa from benign conditions in men undergoing prostate biopsy. **Materials and Methods:** Eighty men who underwent systematic and targeted transrectal prostate biopsy were retrospectively studied. Clinical variables included serum PSA, testosterone, prostate volume, PSA density (PSAD), and the T/PSA ratio. Diagnostic accuracy was assessed using Receiver Operating Characteristic (ROC) curve analysis. Optimal cutoffs were determined using Youden’s index. **Results:** PCa was diagnosed in 53 patients (66.3%). Median T/PSA was significantly lower in PCa versus non-PCa patients (0.46 vs. 0.86; *p* < 0.01). T/PSA showed good diagnostic performance (AUC = 0.75) with an optimal cutoff of 0.81 (sensitivity: 59.3%, specificity: 86.8%). In patients with PSA ≤10 ng/mL, T/PSA retained strong discriminatory ability (AUC = 0.76), with sensitivity and specificity of 82.4% and 72.7%, respectively. Among all parameters, PSAD showed the highest diagnostic accuracy (AUC = 0.813). T/PSA was not significantly associated with Gleason score (*p* = 0.48). **Conclusions:** The T/PSA ratio is a clinically accessible and cost-effective biomarker that may improve PCa risk stratification and reduce unnecessary biopsies, particularly in patients with borderline PSA levels. Although it does not correlate with tumor aggressiveness, its combination with PSAD could enhance diagnostic accuracy in routine clinical practice.

## 1. Introduction

Prostate cancer (PCa) is the second most commonly diagnosed malignancy in men worldwide. While prostate-specific antigen (PSA) testing has improved early detection and reduced mortality by 25%, its low specificity has led to overdiagnosis and overtreatment, particularly in cases of indolent disease [1,2]. Many low-grade tumors, which may never progress, are treated with radical prostatectomy or radiotherapy, increasing the risk of urinary incontinence, erectile dysfunction, and other complications [3]. To mitigate this, researchers are investigating more refined risk stratification tools that could improve biopsy selection and avoid unnecessary treatment [4].

Various alternative biomarkers have been proposed to improve PCa detection. The Prostate Health Index (PHI) and 4Kscore test have demonstrated improved specificity for detecting clinically significant PCa (csPCa) but remain costly and inaccessible in routine clinical practice [5]. Urinary biomarkers, such as PCA3 and SelectMDx, show promise for repeat biopsy decisions but have not been widely integrated into initial screening strategies [6], while multiparametric MRI (mpMRI) has enhanced the detection of csPCa and reduced unnecessary biopsies [7]. Given these limitations, cost-effective, serum-based biomarkers that refine risk stratification in routine practice are needed [8].

There is a debate about the role of testosterone in PCa detection and progression. While early theories suggested that higher testosterone levels promote PCa growth, recent studies have indicated an inverse association between low testosterone and more aggressive disease [9]. Karamanolakis et. al. proposed the testosterone-to-PSA (T/PSA) ratio as a potential biomarker to enhance risk stratification, particularly in men with PSA levels in the diagnostic “gray zone” [10]. Several studies have suggested that lower T/PSA ratios are associated with a higher likelihood of PCa [10,11,12,13]. However, conflicting evidence exists, as studies have found no significant association between T/PSA and PCa risk, suggesting that its clinical utility remains uncertain [14,15,16].

Another promising biomarker is PSA density (PSAD), which adjusts PSA levels for prostate volume. Since PSA elevations may result from benign prostatic hyperplasia (BPH) rather than malignancy, PSAD provides a more specific assessment of cancer risk [17]. Studies by Yusim et al. and Nordström et al. have shown that PSAD outperforms PSA alone in predicting clinically significant PCa, supporting its use in biopsy decision-making [7,18]. Tafuri et al. further demonstrated that PSAD correlates with ISUP grade group, making it a valuable tool for pre-biopsy risk assessment [19].

Given the need for improved risk stratification, a combined approach integrating new parameters and traditional PSA measures may offer better diagnostic accuracy while minimizing unnecessary biopsies [4]. This study aims to evaluate the diagnostic performance of T/PSA in distinguishing PCa from benign conditions, assessing their cost-effectiveness and clinical applicability in reducing overdiagnosis and overtreatment. By analyzing their predictive accuracy alongside PSA, we seek to determine whether these markers can improve biopsy selection and enhance early detection of clinically significant disease.

## 2. Materials and Methods

### 2.1. Study Design and Patient Selection

This retrospective study was conducted at the Urology Department of the University General Hospital of Alexandroupolis. The study included 80 male patients who underwent systematic and targeted transrectal prostate biopsy between January 2023 and December 2024. Eligible participants were men over the age of 50 with serum PSA levels exceeding 2.5 ng/mL at the time of evaluation. In cases of a negative digital rectal examination (DRE), biopsy was performed based on elevated PSA levels and/or suspicious findings on multiparametric MRI with a PI-RADS score of ≥3. The following parameters were assessed for all patients: age, serum PSA levels, serum testosterone levels, testosterone-to-PSA ratio, prostate volume, PSA density, digital rectal examination findings, multiparametric MRI results with PI-RADS classification, and histopathological findings, including Gleason score in patients diagnosed with prostate cancer.

Patients were excluded if they were receiving pharmacological agents known to reduce PSA levels, such as finasteride or dutasteride, or if they had a prior diagnosis of prostate cancer. Additionally, individuals with symptomatic or asymptomatic urinary tract infections preceding their assessment were excluded. Patients who had undergone medical procedures capable of influencing PSA values, including digital rectal examination, indwelling Foley catheterization, or cystoscopic evaluation within the two weeks before PSA measurement, were also excluded. Finally, cases with incomplete medical records were not considered for analysis.

All patients were informed and consented before the procedure. All procedures performed in this study involving human participants were in accordance with the ethical standards of the institutional and national research committee and with the 1964 Helsinki Declaration and its later amendments or comparable ethical standards. Given its retrospective nature and the anonymization of patient data, the requirement for obtaining individual informed consent was waived.

### 2.2. Blood Sample Collection and Hormonal Assessment

Venous blood samples were collected from all participants between 08:00 and 10:00 h to ensure consistency in hormonal measurements and to minimize the impact of circadian variations. Samples were processed within two hours of collection. Serum testosterone levels were quantified using an electrochemiluminescence immunoassay (ECLIA) performed on a COBAS E-602 analyzer (Roche Diagnostics, Basel, Switzerland), utilizing a dedicated testosterone assay kit specific to this platform. The reference range for serum testosterone was defined as 2.8–8.0 ng/mL.

### 2.3. Prostate Biopsy Procedure

All included patients underwent systematic transrectal prostate biopsy under ultrasound guidance, following a standardized 12-core sampling scheme. The procedure was performed with the patient in the left lateral decubitus position under local anesthesia. Prior to biopsy, digital rectal examination and transrectal ultrasonography (TRUS) were performed to assess prostate volume and identify any suspicious lesions. In addition to systematic sampling, targeted biopsies were performed in cases where multiparametric MRI revealed suspicious prostate lesions (PI-RADS ≥ 3), with three additional cores obtained from these areas. The biopsy procedure was executed using an 18-gauge biopsy needle (Magnum™, Bard Biopsy Systems, Tempe, AZ, USA), guided by an ultrasound scanner (BK2202, BK Medical, Herlev, Denmark) equipped with a 10/7.5 MHz probe. Tissue specimens were individually labeled, fixed, and processed according to standardized histopathological protocols, with Gleason score assessment performed for all prostate cancer cases.

### 2.4. Statistical Analysis

Statistical analyses were conducted using SPSS version 23.0 (SPSS Inc., Chicago, IL, USA). Continuous variables, including patient age, serum PSA levels, serum testosterone levels, T/PSA ratio, prostate volume, and PSAD, were assessed for normality. Since these variables did not follow a normal distribution, they were summarized using medians and interquartile ranges (IQRs).

Comparisons between groups were performed using the Mann–Whitney U test. ROC curve analysis was conducted to evaluate the diagnostic performance of T/PSA ratio, PSA, prostate volume, PSAD, and testosterone in distinguishing PCa from non-PCa cases. The optimal T/PSA cutoff value was determined using Youden’s J statistic. Subgroup analysis was performed for patients with PSA ≤ 10 ng/mL. Logistic regression analysis was used to determine the odds ratios (ORs) and 95% confidence intervals (CIs) for predictive variables.

## 3. Results

### 3.1. Patient Characteristics

A total of 80 patients who underwent prostate biopsy were included in the study. The median age of the cohort was 70 years (IQR: 65–74.25). The median PSA level was 8.18 ng/mL (IQR: 6.72–11.75), and the median testosterone level was 5.00 ng/mL (IQR: 3.51–6.35). The median T/PSA ratio was 0.55 (IQR: 0.37–0.85). Prostate cancer was diagnosed in 53 patients (66.25%), with 29 (54.7%) classified as Gleason score 6 and 24 (45.3%) as Gleason score ≥7. Among the patients who were diagnosed with PCa, 90.5% had abnormal DRE defined as the presence of a hard or indurated prostate area, and all patients with Gleason scores of 7(4 + 3), 8, and 9 had abnormal DRE.

Multiparametric MRI findings were available for all patients. Among them, 29 (36.25%) had no suspicious lesions, 13 (16.25%) had PI-RADS 3 lesions, 25 (31.25%) had PI-RADS 4 lesions, and 13 (16.25%) had PI-RADS 5 lesions. The presence of high PI-RADS scores (4 and 5) was strongly associated with PCa diagnosis. Table 1 summarizes the patient characteristics of this study.

### 3.2. Comparison of Clinical Parameters Between Prostate Cancer and Non-Cancer Groups

Patients diagnosed with PCa were significantly older than those without cancer (median: 72 vs. 69 years, *p* = 0.052). Median testosterone levels were significantly lower in the PCa group (4.16 ng/mL, IQR: 2.93–5.77) compared to the non-cancer group (6.02 ng/mL, IQR: 4.84–6.80, *p* < 0.01). PSA levels were higher in the PCa group (8.49 ng/mL, IQR: 7.15–14.00) compared to the non-cancer group (7.40 ng/mL, IQR: 6.30–9.61, *p* = 0.046). The T/PSA ratio was significantly lower in the PCa group (0.46, IQR: 0.30–0.73) compared to the non-cancer group (0.86, IQR: 0.50–1.06, *p* < 0.01). Prostate volume was significantly lower in PCa patients (42 cc, IQR: 35–55) than in non-cancer patients (55 cc, IQR: 42.5–65, *p* = 0.01). PSA density was significantly higher in PCa patients (0.20 ng/mL/cc, IQR: 0.15–0.28) compared to non-cancer patients (0.13 ng/mL/cc, IQR: 0.12–0.16, *p* < 0.01). Table 2 summarizes the characteristics of the patients in the non-cancer group along with the PCa group.

### 3.3. Diagnostic Performance of T/PSA

**Figure 1:**
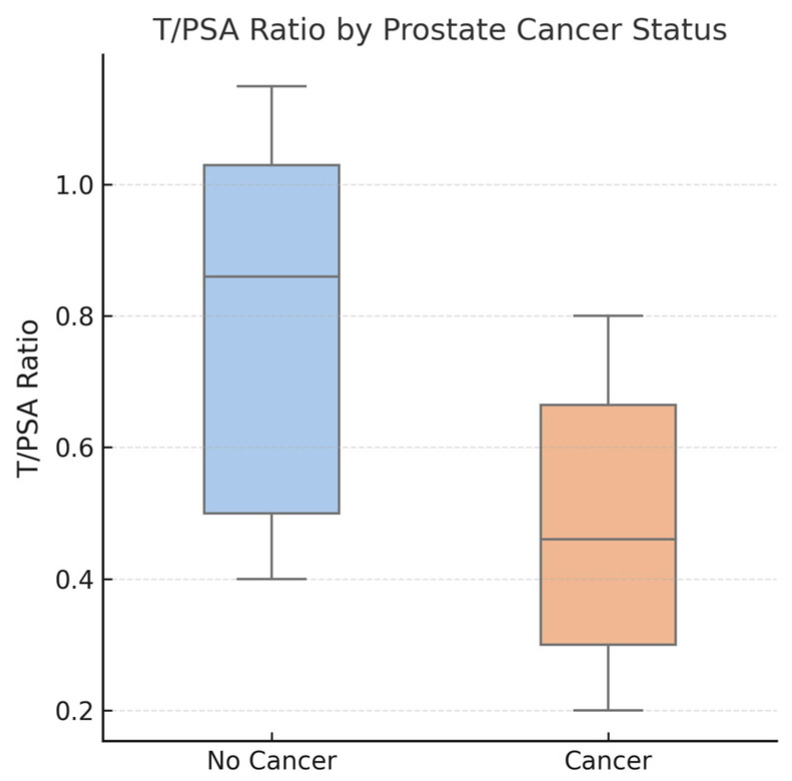
Boxplot comparing testosterone-to-PSA (T/PSA) ratios between patients diagnosed with prostate cancer (PCa) and those without cancer. The median T/PSA ratio was significantly lower in the PCa group (median: 0.46) compared to the non-cancer group (median: 0.86), with *p* < 0.01. Boxes represent the interquartile range (IQR), the horizontal line indicates the median, and individual data points are shown with jitter. The lower T/PSA ratio in the PCa group suggests its potential diagnostic utility.

**Figure 2:**
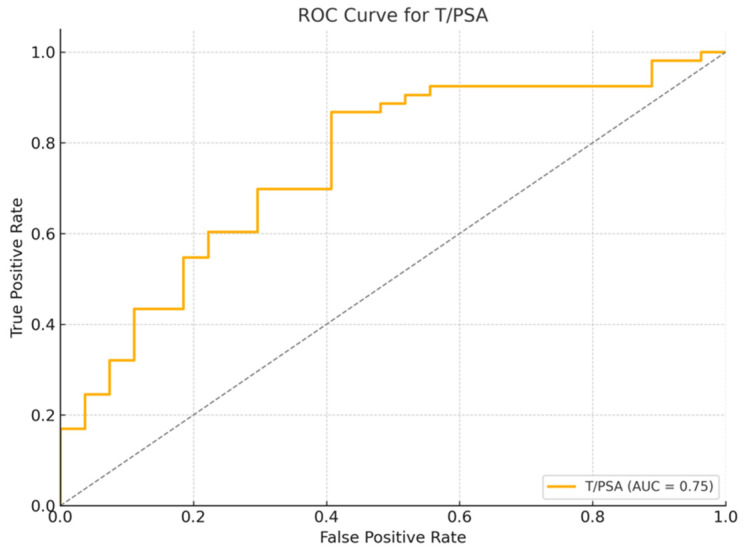
Receiver Operating Characteristic (ROC) curve for T/PSA ratio in patients with and without prostate cancer. The area under the curve (AUC) is 0.75, indicating good discrimination between cancer and non-cancer cases.

ROC curve analysis of the T/PSA ratio for distinguishing PCa from non-PCa cases demonstrated an AUC of 0.75, indicating good discriminatory ability. The optimal cutoff value for T/PSA, determined using Youden’s J statistic, was 0.81 (OR = 0.10, 95% CI: 0.03–0.32). This threshold yielded a sensitivity of 59.3% and a specificity of 86.8%. Boxplot and ROC curves of T/PSA in patients with and without PCa can be seen in Figure 1 and Figure 2, respectively.

### 3.4. Diagnostic Performance of Other Parameters

**Figure 3:**
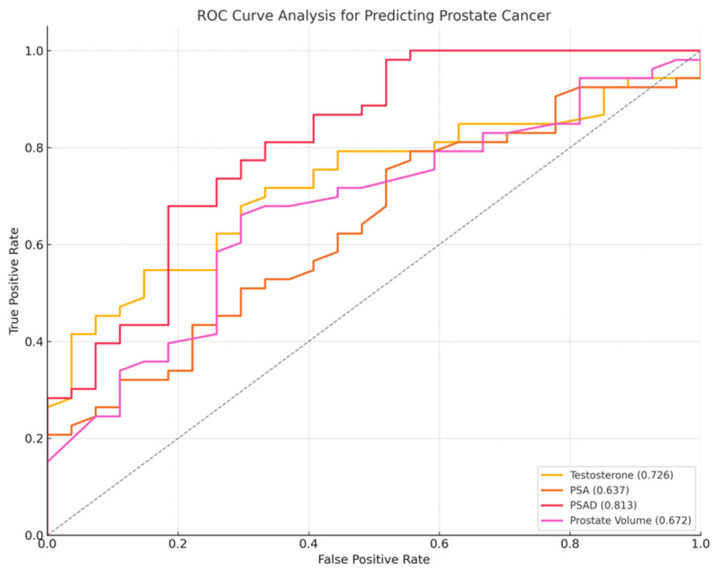
ROC curve for prostate cancer diagnosis, PSA (orange line), PSAD (red line), testosterone (yellow line), prostate volume (purple line). PSAD demonstrated the highest diagnostic performance, with an AUC of 0.813, followed by PSA (AUC = 0.637), testosterone (AUC = 0.726), and prostate volume (AUC = 0.672). Higher values of testosterone and prostate volume were more associated with non-cancer cases.

A Receiver Operating Characteristic (ROC) curve analysis was performed to assess the diagnostic performance of testosterone, PSA, prostate volume, and PSAD in differentiating between prostate cancer and non-cancer cases (Figure 3).

Among the examined biomarkers, PSAD demonstrated the highest AUC (0.813), followed by testosterone (AUC = 0.726), prostate volume (AUC = 0.672), and PSA (AUC = 0.637). PSA exhibited high sensitivity (98.1%) but very poor specificity, while PSAD provided the best balance of sensitivity (77.4%) and specificity (66.7%) at a cutoff of 0.16 ng/mL/cc (OR = 40.70 × 10^6^, 95% CI: 744.08–2.23 × 10^12^, *p* < 0.0001). Higher PSAD values were the strongest predictor of prostate cancer (*p* < 0.01). Lower testosterone levels (OR = 0.738, 95% CI: 0.575–0.947, *p* < 0.01) and smaller prostate volumes (OR = 0.977, 95% CI: 0.955–0.999, *p* = 0.04) were significantly associated with increased prostate cancer risk.

To evaluate whether the T/PSA ratio remains an independent predictor of prostate cancer after adjusting for potential confounding variables, a multivariate logistic regression analysis was performed, including T/PSA, age, PSAD, and prostate volume as covariates. The analysis demonstrated that the T/PSA ratio retained statistical significance (OR = 0.10, 95% CI: 0.01–0.79, *p* = 0.029), indicating that lower T/PSA values are independently associated with an increased likelihood of prostate cancer. 

### 3.5. Subgroup Analysis of Patients with PSA ≤ 10 ng/mL

A subset of 56 patients with PSA ≤ 10 ng/mL was analyzed separately. Among them, 34 were diagnosed with PCa and 22 were cancer-free. Significant differences were observed in testosterone levels (PCa: 4.45 ng/mL, IQR: 2.95–5.76 vs. non-PCa: 6.12 ng/mL, IQR: 5.55–6.86, *p* = 0.01), T/PSA ratio (PCa: 0.61, IQR: 0.45–0.78 vs. non-PCa: 0.92, IQR: 0.71–1.08, *p* = 0.01), prostate volume (PCa: 40 cc, IQR: 33–47 vs. non-PCa: 54.5 cc, IQR: 40–61.75, *p* < 0.01), and PSAD (PCa: 0.18 ng/mL/cc, IQR: 0.15–0.28 vs. non-PCa: 0.12 ng/mL/cc, IQR: 0.11–0.13, *p* < 0.01). PSA levels, however, were not significantly different between the two groups (*p* = 0.411).

**Figure 4:**
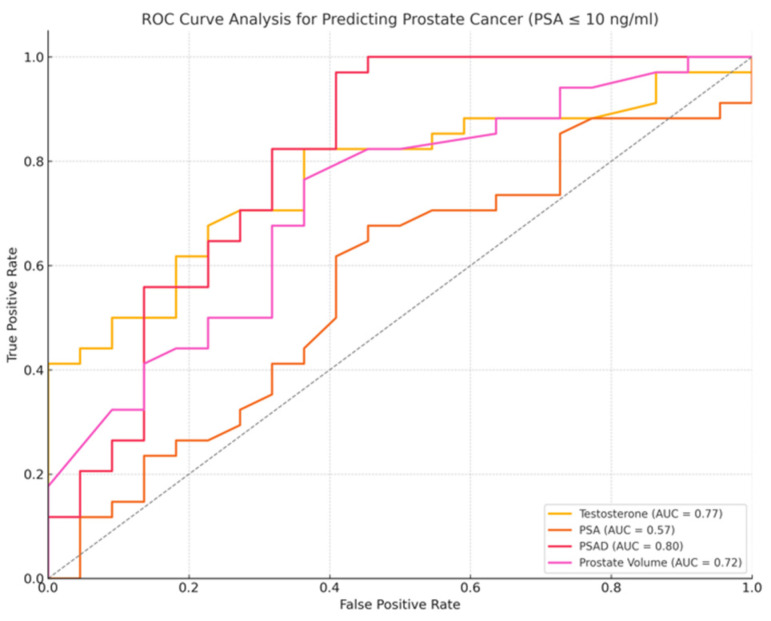
ROC curve for prostate cancer diagnosis in patients with PSA ≤ 10 ng/mL, PSA (orange line), PSAD (red line), testosterone (yellow line), prostate volume (purple line). PSAD demonstrated the highest diagnostic performance, with an AUC of 0.80, followed by PSA (AUC = 0.57), testosterone (AUC = 0.77), and prostate volume (AUC = 0.72). Higher values of testosterone and prostate volume were more associated with non-cancer cases.

**Figure 5:**
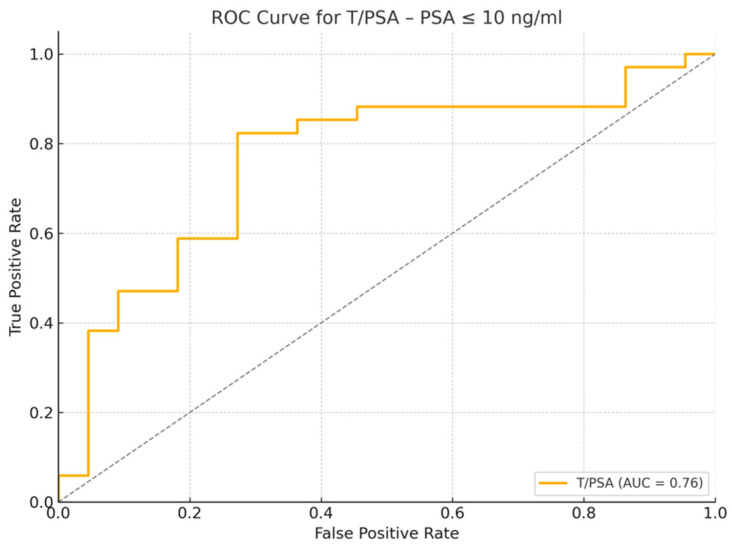
Receiver Operating Characteristic (ROC) curve for T/PSA Ratio in patients with PSA ≤ 10 ng/mL. The area under the curve (AUC) is 0.76, indicating good discrimination between cancer and non-cancer cases.

**Figure 6:**
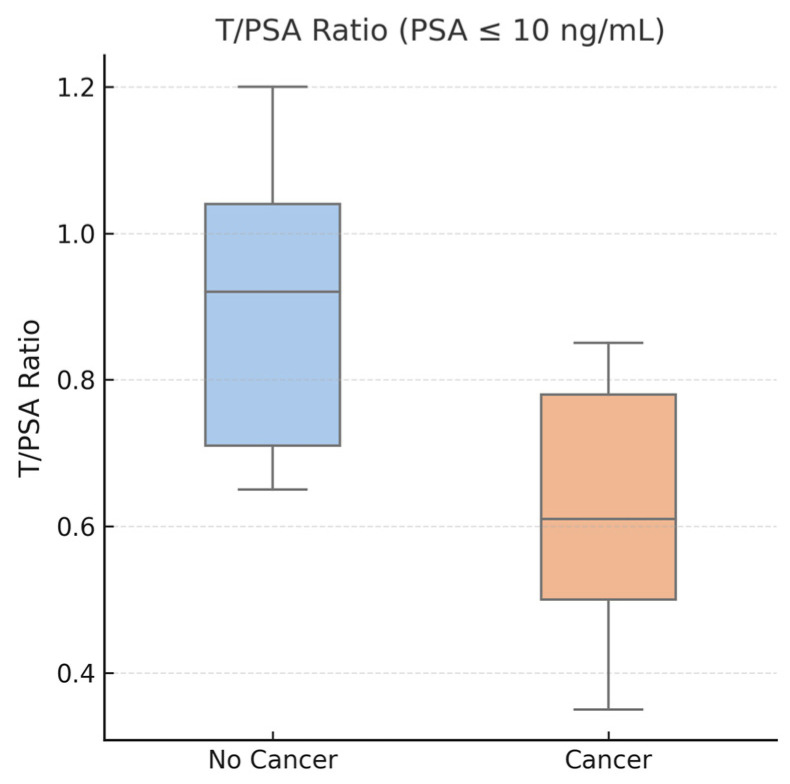
Boxplot showing T/PSA ratios in patients with PSA ≤ 10 ng/mL, stratified by cancer diagnosis. The median T/PSA ratio was significantly lower in the PCa group (median: 0.61) compared to non-cancer patients (median: 0.92), with *p* = 0.01. This figure highlights the potential diagnostic value of T/PSA in men within the PSA “gray zone”, where PSA alone lacks specificity.

T/PSA had the highest specificity of 72.7% with a sensitivity of 82.4% at a cutoff of 0.8 (OR: 0.10, 95% CI: 1.83–63.39, *p* = 0.008), while PSAD demonstrated the highest sensitivity of 97.1% at a cutoff of 0.13 ng/mL/cc (OR = 3.56 × 10^−10^, 95% CI: 9.10 × 10^−28^–1.40 × 10^8^, *p* = 0.293). Figure 4, Figure 5 and Figure 6 demonstrate the ROC curve analysis and the boxplot of T/PSA, while Table 3 summarizes the characteristics of the patients with PSA ≤ 10 ng/mL with and without prostate cancer. Boxplot and ROC curves of T/PSA and for the other parameters in patients with and without PCa and PSA ≤ 10 ng/mL can be seen in Figure 4, Figure 5 and Figure 6.

A separate multivariate logistic regression analysis was performed for the subgroup of patients with PSA ≤ 10 ng/mL to assess whether the T/PSA ratio retains its predictive value in this clinically relevant cohort. The model included T/PSA, age, PSA density, and prostate volume as covariates. The T/PSA ratio remained a statistically significant independent predictor of prostate cancer (OR = 0.02, 95% CI: 0.001–0.41, *p* = 0.010), further supporting its diagnostic relevance in patients within the PSA “gray zone”.

### 3.6. Association of T/PSA with Gleason Score

**Figure 7:**
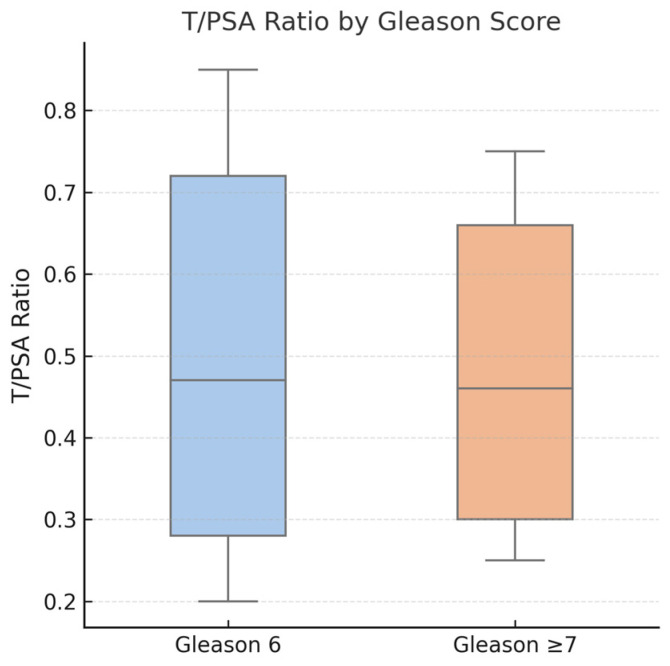
Boxplot comparing T/PSA ratios in patients with prostate cancer, stratified by Gleason score. No statistically significant difference was observed between patients with Gleason 6 score (median: 0.47) and those with Gleason score ≥ 7 (median: 0.46), *p* = 0.48. These findings suggest that while T/PSA may aid in cancer detection, it does not correlate with tumor aggressiveness.

Patients were further stratified by Gleason score (Gleason 6 vs. ≥7). The median T/PSA ratio in the Gleason 6 group was 0.47 (IQR: 0.28–0.72), while in the Gleason ≥7 group, it was 0.46 (IQR: 0.30–0.66). This difference was not statistically significant (*p* = 0.48), suggesting that while T/PSA is a potential pre-biopsy biomarker, it does not seem to correlate with prostate cancer aggressiveness. Table 4 summarizes the characteristics of patients with prostate cancer and Gleason score, while Figure 7 demonstrates the boxplot comparing T/PSA ratios in patients with prostate cancer.

## 4. Discussion

The testosterone-to-PSA ratio calculated in this study was significantly lower in patients diagnosed with prostate cancer compared to those without cancer (median T/PSA 0.46 vs. 0.86, *p* < 0.01). These findings align closely with earlier studies, which reported significantly lower T/PSA ratios in PCa cases, reinforcing the potential role of T/PSA as a useful biomarker in pre-biopsy screening, despite there being no consensus on the cutoff values, with varying levels of sensitivity and specificity [10,11,12,13,20]. The calculated cutoff value was 0.81 (OR = 0.10, 95% CI: 0.03–0.32), with a sensitivity of 59.3% and a specificity of 86.8%. The high specificity suggests it may effectively reduce unnecessary biopsies by correctly identifying men without cancer. However, its moderate sensitivity indicates a risk of missing some cases of clinically significant prostate cancer. This trade-off should be carefully considered in clinical applications. However, the literature remains divided, with several studies reporting no significant differences in T/PSA between patients with PCa and those with benign conditions [14,15,16,21]. These discrepancies likely arise from variations in study populations, diagnostic thresholds, and methodologies.

Subgroup analysis of patients with PSA ≤ 10 ng/mL demonstrated that T/PSA retained significant discriminatory power, with a median T/PSA of 0.61 in patients with PCa compared to 0.92 in those without PCa (*p* = 0.01). These findings highlight the potential utility of T/PSA in patients with moderately elevated PSA, a cohort in which conventional PSA testing lacks sufficient specificity. The determined T/PSA cutoff of 0.8 (OR: 0.10, 95% CI: 1.83–63.39, *p* = 0.008) yielded a sensitivity of 82.4% and a specificity of 72.7%, indicating that it may help avoid unnecessary invasive procedures without compromising cancer detection. Consistent with previous literature, our results indicate that T/PSA values are significantly lower in patients with PCa. However, the variability in cutoff values across different studies underscores the complexity of establishing universally applicable thresholds [11,13].

The multivariate logistic regression analyses further underscored the independent diagnostic value of the T/PSA ratio. In the full patient cohort, T/PSA remained a statistically significant predictor of prostate cancer after adjusting for age, PSA density (PSAD), and prostate volume (OR = 0.10, 95% CI: 0.01–0.79, *p* = 0.029). Notably, in the subgroup of patients with PSA ≤ 10 ng/mL, the T/PSA ratio continued to demonstrate independent predictive value (OR = 0.02, 95% CI: 0.001–0.41, *p* = 0.010), reinforcing its relevance in men with borderline PSA levels. These findings suggest that the T/PSA ratio provides additional diagnostic information beyond conventional clinical variables and may be particularly valuable in refining biopsy decisions in patients within the PSA “gray zone”.

Despite its diagnostic potential, T/PSA did not seem to correlate with tumor aggressiveness in our analysis, reflected by similar median values between the Gleason 6 and Gleason ≥7 groups (0.47 vs. 0.46, *p* = 0.48). This aligns with studies by Schwarzman et al. and Morote et al., who similarly reported no prognostic association between T/PSA and PCa severity [14,15]. Hence, while it seems valuable for initial PCa detection, T/PSA alone may not effectively guide prognosis or aggressiveness evaluation. Therefore, the clinical utility of T/PSA should be interpreted primarily within the context of pre-biopsy decision-making rather than post-diagnostic risk stratification. Its role lies in aiding the decision to proceed with biopsy in men with equivocal PSA values, rather than predicting disease severity or guiding treatment intensity.

This study’s findings also emphasize PSA density (PSAD) as a highly effective diagnostic marker, with a notable area under the curve (AUC) of 0.813, outperforming PSA alone. This observation is consistent with previous research. Yusim et al. reported an optimal PSAD cutoff of 0.20 ng/mL^2^ with a sensitivity of 70% and a specificity of 79% [18], whereas Nordström et al. highlighted PSAD ≤ 0.07 ng/mL^2^ as a potential threshold to safely avoid biopsies, reducing unnecessary procedures by nearly 20% [7]. Additionally, Gürbüz et al. and Park et al. further corroborated the utility of PSAD in enhancing specificity [17,22]. Our optimal PSAD cutoff (0.15 ng/mL^2^) demonstrated strong performance, with sensitivity and specificity of 81.3% and 73.6%, respectively, solidifying PSAD’s clinical importance in differentiating PCa from benign prostatic hyperplasia. Notably, 93% of patients diagnosed with a Gleason score of 7 (4 + 3), 8, or 9 had a PSAD ≥ 0.2 ng/mL/cc.

Significant associations were further revealed between lower testosterone levels, smaller prostate volumes, and an increased risk of prostate cancer. These inverse relationships between serum testosterone and PCa risk are consistent with findings from previous studies [19,22,23]. However, research on the relationship between androgen concentrations, serum PSA levels, and PCa risk has produced inconsistent and often conflicting results. While some studies have reported reduced testosterone levels in patients diagnosed with PCa [10,17,22], a larger body of evidence has found no significant correlation between serum testosterone levels and PCa [9,14,15,16].

The diagnostic value of T/PSA and PSAD may be enhanced by incorporating them into a structured diagnostic algorithm, particularly for patients with intermediate risk profiles. Patients, particularly with PSA levels in the diagnostic “gray zone”, with a normal digital rectal examination, multiparametric MRI PI-RADS ≤ 3, and favorable T/PSA > 0.81 values may safely avoid biopsy. Additionally, those with intermediate T/PSA levels (0.6–0.81) and PSAD < 0.16 ng/mL/cc, in combination with a normal DRE, could reasonably defer immediate biopsy, reducing unnecessary procedures. In contrast, patients with elevated PSAD, abnormal DRE, or suspicious mpMRI findings (PI-RADS ≥ 4) should be prioritized for biopsy. This stepwise, multimodal approach aligns with the recommendations of Williams et al., who advocate for integrating clinical and biomarker-based assessments to optimize risk stratification [4].

Although advanced biomarkers like the Prostate Health Index (PHI), 4Kscore test, PCA3, and SelectMDx have demonstrated improved specificity, their clinical adoption remains limited due to high costs and limited accessibility [5,6,24]. By contrast, T/PSA and PSAD represent accessible, economically viable tools for initial screening and biopsy stratification. The integration of these cost-effective biomarkers with advanced imaging modalities such as mpMRI could represent an optimal diagnostic paradigm, potentially maximizing clinical benefit by minimizing overdiagnosis and overtreatment.

### Limitations

This study has several limitations that should be acknowledged. The retrospective design introduces potential selection bias, as data were extracted from medical records rather than collected prospectively. As a result, unmeasured confounding variables may have influenced the observed associations. Additionally, the sample size of 80 patients, while providing valuable preliminary insights, remains relatively small. Larger, multicenter studies are needed to validate these findings and improve their generalizability.

The study population was limited to patients undergoing systematic and targeted transrectal prostate biopsy at a single institution. This may restrict the applicability of the results to broader populations with varying clinical practices, biopsy techniques, and patient demographics. Furthermore, serum testosterone variability poses a challenge in hormone-related studies. Although blood samples were collected in the morning (08:00–10:00 AM) to minimize diurnal fluctuations, individual differences due to comorbidities or medication use may still have affected the accuracy of T/PSA measurements.

While this study highlights the diagnostic potential of T/PSA and PSAD, the lack of standardized cutoff values across different studies complicates their clinical adoption. Establishing universally accepted thresholds through larger prospective trials is essential for their consistent and reliable application in clinical practice.

To address these limitations, future prospective studies with larger, multicenter cohorts are needed to confirm the diagnostic accuracy of T/PSA and PSAD. Additionally, incorporating long-term follow-up data will help assess their prognostic value in disease progression and treatment outcomes. Integrating these markers with emerging molecular and imaging biomarkers may further enhance risk stratification, ultimately improving biopsy decision-making and reducing unnecessary interventions.

## 5. Conclusions

T/PSA was significantly lower in PCa patients, suggesting its potential clinical utility in distinguishing PCa from benign conditions. However, no significant association was found between T/PSA and tumor aggressiveness. PSAD demonstrated the highest diagnostic performance, with values ≥ 0.2 ng/mL/cc strongly associated with high-grade PCa (Gleason scores of 7 (4 + 3), 8, and 9), reinforcing its role as a predictor of clinically significant disease. The inverse association between serum testosterone levels and PCa risk aligns with some previous studies, though further research is needed to clarify its diagnostic significance. Although it does not correlate with tumor aggressiveness and should not be used for prognostication, its integration alongside traditional measures such as PSAD, DRE, and mpMRI may help refine biopsy decisions. In particular, its utility appears most pronounced in patients with PSA ≤ 10 ng/mL, where conventional markers often lead to diagnostic uncertainty. Large prospective multicenter studies are required to validate these findings, standardize cutoff values, and assess their role in long-term risk stratification and treatment planning.

## Figures and Tables

**Table 1:** Patient characteristics.

Variables	Median (IQR)
Age (years)	70 (65–74.25)
Testosterone (ng/mL)	5 (3.51–6.35)
PSA (ng/mL)	8.18 (6.72–11.75)
Testosterone/PSA, (T/PSA)	0.55 (0.37–0.85)
Prostate volume (cc)	46 (35–58.5)
PSAD (ng/mL/cc)	0.17 (0.13–0.25)
Variables	n (%)
mpMRI	
Without	29 (36.25)
Pirads 3	13 (16.25)
Pirads 4	25 (31.25)
Pirads 5	13 (16.25)
DRE	
Normal	36 (45)
Abnormal	44 (55)
Prostate Cancer	
No	27 (33.75)
Yes	53 (66.25)
Gleason score = 6	29 (54.7)
Gleason score ≥ 7	24 (45.3)

**Table 2:** Characteristics of patients without and with prostate cancer.

	Without Prostate Cancer	With Prostate Cancer	
Variables	Median (IQR)	Median (IQR)	*p*-Value
Age (years)	69 (61.5–72.5)	72 (68–75)	0.052
Testosterone (ng/mL)	6.02 (4.84–6.8)	4.16 (2.93–5.77)	<0.01
PSA (ng/mL)	7.4 (6.3–9.61)	8.49 (7.15–14)	0.046
Testosterone-to-PSA (T/PSA)	0.86 (0.5–1.06)	0.46 (0.3–0.73)	<0.01
Prostate volume (cc)	55 (42.5–65)	42 (35–55)	0.01
PSAD (ng/mL/cc)	0.13 (0.12–0.16)	0.2 (0.15–0.28)	<0.01

**Table 3:** Characteristics of patients without and with prostate cancer and PSA ≤ 10 ng/mL.

	Without Prostate Cancer	With Prostate Cancer	
Variables	Median (IQR)	Median (IQR)	*p*-Value
Age (years)	66.5 (60.25–71.5)	71 (66.25–75)	0.058
Testosterone (ng/mL)	6.12 (5.55–6.86)	4.45 (2.95–5.76)	0.01
PSA (ng/mL)	6.77 (5.63–8.14)	7.37 (6.28–8.34)	0.411
Testosterone-to-PSA, (T/PSA)	0.92 (0.71–1.08)	0.61 (0.45–0.78)	0.01
Prostate volume (cc)	54.5 (40–61.75)	40 (33–47)	<0.01
PSAD (ng/mL/cc)	0.12 (0.11–0.13)	0.18 (0.15–0.28)	<0.01

**Table 4:** Characteristics of patients with prostate cancer and Gleason score.

	Gleason Score 6	Gleason Score ≥ 7	
Variables	Median (IQR)	Median (IQR)	*p*-Value
Testosterone-to-PSA, (T/PSA)	0.47 (0.28–0.72)	0.46 (0.3–0.66)	0.48

## Data Availability

The data are available from the corresponding author upon reasonable request.
